# Uric Acid Levels Can Predict Metabolic Syndrome and Hypertension in Adolescents: A 10-Year Longitudinal Study

**DOI:** 10.1371/journal.pone.0143786

**Published:** 2015-11-30

**Authors:** Hai-Lun Sun, Dee Pei, Ko-Huang Lue, Yen-Lin Chen

**Affiliations:** 1 Department of Pediatrics, Chung Shan Medical University Hospital, School of Medicine, Chung Shan Medical University, Taichung, Taiwan; 2 Department of Internal Medicine, Cardinal Tien Hospital, School of Medicine, Fu-Jen Catholic University, New Taipei, Taiwan; 3 Department of Pathology, Cardinal Tien Hospital, School of Medicine, Fu-Jen Catholic University, New Taipei, Taiwan; Medical University Innsbruck, AUSTRIA

## Abstract

The relationships between uric acid and chronic disease risk factors such as metabolic syndrome, type 2 diabetes mellitus, and hypertension have been studied in adults. However, whether these relationships exist in adolescents is unknown. We randomly selected 8,005 subjects who were between 10 to 15 years old at baseline. Measurements of uric acid were used to predict the future occurrence of metabolic syndrome, hypertension, and type 2 diabetes. In total, 5,748 adolescents were enrolled and followed for a median of 7.2 years. Using cutoff points of uric acid for males and females (7.3 and 6.2 mg/dl, respectively), a high level of uric acid was either the second or third best predictor for hypertension in both genders (hazard ratio: 2.920 for males, 5.222 for females; p<0.05). However, uric acid levels failed to predict type 2 diabetes mellitus, and only predicted metabolic syndrome in males (hazard ratio: 1.658; p<0.05). The same results were found in multivariate adjusted analysis. In conclusion, a high level of uric acid indicated a higher likelihood of developing hypertension in both genders and metabolic syndrome in males after 10 years of follow-up. However, uric acid levels did not affect the occurrence of type 2 diabetes in both genders.

## Introduction

The clustering of overweight, high blood pressure, high blood glucose and dyslipidemia has previously been reported, which is important because of the close relationships with cardiovascular diseases (CVD) and type 2 diabetes (DM) [1,2]. Due to the ‘endemic’ nature of these modern diseases, the way to identifying subjects at risks has become increasingly important. In 2003, the World Health Organization published the definition of metabolic syndrome (MetS) [3]. Currently, the International Diabetes Federation consensus report for definition of the MetS for children and adolescents in 2007 is the most well recognized [4]. Nowadays in Asia, life style has been dramatically westernized. The consequence of this change is the increase of obesity. Indirectly affected by this phenomenon, the incidence of MetS and hyperuricemia also become higher [5,6].

Before the definition of MetS was made, particularly in adults, most studies used the occurrence of CVD and/or type 2 DM as their endpoints. Although these endpoints are easily observed in older patients, they are not easily observed in younger cohorts as it would take years for young adults to reach these endpoints. Other than the originally proposed criteria for MetS, many other non-traditional risk factors have also been identified such as increased hematogram result, liver enzymes and liver echogenicity [7,8]. Among them, uric acid (UA) has also been well studied. We previously performed a longitudinal cohort study and found that serum UA was correlated with MetS components in adolescents [9]. An elevated level of UA had also been found to be associated with type 2 DM in adults [10,11]. Although the relationships between UA and MetS components have been studied extensively in adults and the elderly, whether these relationships are the same in adolescents is unknown. To the best of our knowledge, no large-scale cohort study has focused on this topic. Therefore, in the present study, 5,748 adolescent were enrolled and followed for a median of 7.2 years in order to elucidate the relationships between UA and MetS, hypertension and type 2 DM.

## Methods

### Study population

We randomly selected 8,005 subjects aged between 10 to 15 years old from 1999 to 2008 from MJ Health Screening Centers, a privately-owned chain of clinics located throughout Taiwan which provide regular health examinations to their members. The informed consent was obtained from each. Due to the participants were all under the age of 18, the written informed consent was obtained from the next of kin, caretakers, or guardians on behalf of the children enrolled in the current study. The study protocol was approved by the institutional review board of both Cardinal Tien Hospital (CTH IRB) and MJ Health Screening Center (MHSC IRB), and the data were provided for research purposes only.

They were selected according to the following exclusion criteria:

Step 1: Excluded 1,935 subjects with only one visit.
Step 2: Excluded 247 subjects with missing data of MetS components or UA.
Step 3: Excluded 75 subjects with the history of type 1 DM and on medications known to affect MetS components or serum UA levels.

**Fig 1:**
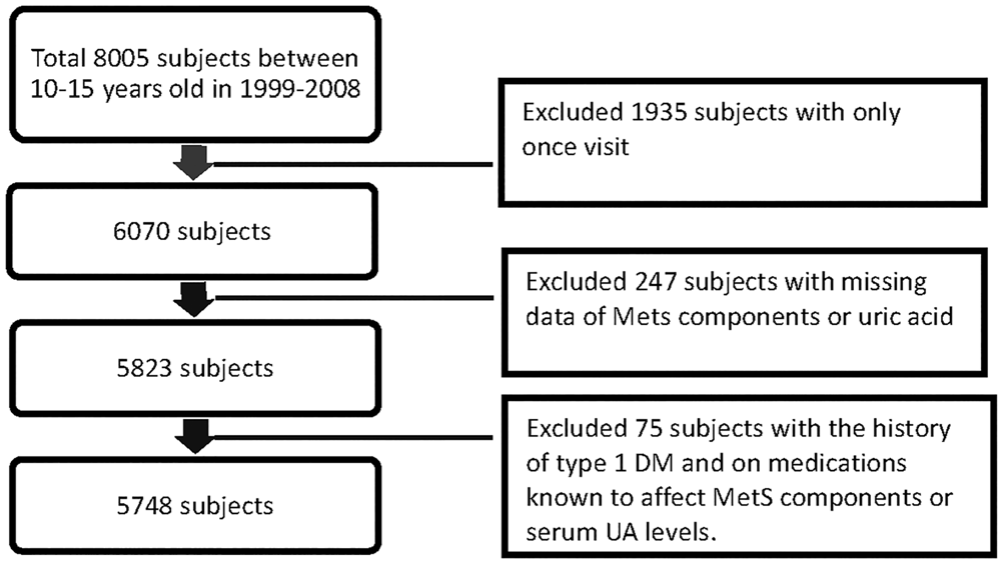
Description of the study population.

Finally, the remaining 5,748 subjects were enrolled as the study cohort. (Fig 1)

There were two parts to the study design. First, a cross-sectional designed observation, the main purpose of which was to identify the optimal cut-off values for UA to predict the future development of MetS using receiver operating characteristic curves (ROC). Second, 445 subjects were excluded due to having MetS at baseline and the remaining 5,303 subjects without MetS were followed for 10 years (median 7.2 years). The purpose of the second part of the study was to confirm, by using the aforementioned cut-off value, whether a higher UA level was associated with higher incidence rates of MetS, hypertension and type 2 DM.

### Anthropometric measurements and general data

A standard protocol for health checkups was followed at the MJ clinics. The senior nursing staff used a questionnaire to obtain the subjects’ medical history, including any current medications and complete physical examinations were then performed. Waist circumference (WC) was measured horizontally at the level of the natural waist, which was identified as the level at the hollow molding of the trunk when the trunk was laterally concave. Body mass index (BMI) was calculated as the subject’s body weight (kg) divided by the square of the subject’s height (m). Both systolic blood pressure (SBP) and diastolic blood pressure (DBP) were measured by the nursing staff using a standard mercury sphygmomanometer fitted on the right arm of each subject when seated. After the subject had fasted for 10 hours, blood samples were drawn from the antecubital vein for biochemical analysis. Plasma was separated from the blood within 1 hour and stored at -30°C until analysis for fasting plasma glucose (FPG) and lipid profiles. FPG was detected using a glucose oxidase method (YSI 203 glucose analyzer, Scientific Division, Yellow Springs Instruments, Yellow Springs, OH). Total cholesterol and triglycerides (TG) were measured using the dry, multilayer analytical slide method in a Fuji Dri-Chem 3000 analyzer (Fuji Photo Film, Minato-Ku, Tokyo, Japan). Serum high-density lipoprotein cholesterol (HDL-C) and low-density lipoprotein cholesterol (LDL-C) concentrations were analyzed using an enzymatic cholesterol assay following dextran sulfate precipitation. Hemoglobin was measured with an Abbott Cell Dyn 3000 hematology analyzer (Abbott Laboratories, Abbott Park, IL, USA). Serum UA levels were measured using an uricase-based method with a Hitachi 7150 automatic biochemical analyzer (Hitachi, Tokyo, Japan).

### Definition of metabolic syndrome

The International Diabetes Federation consensus definition of MetS in children and adolescents was used to define MetS in this study [4]. The subjects who had three or more of the following abnormalities were diagnosed as MetS: abdominal obesity (WC ≥ 90^th^ percentile), TG ≥ 150 mg/dL, HDL-C < 40 mg/dL, hypertension (SBP ≥ 130 and DBP ≥ 85 mmHg), and FPG concentration ≥ 100 mg/dL.

### Statistical analysis

The data in this study were presented as mean ± standard deviation. Dataset used in this study is given in S1 Dataset. All data were tested for normal distribution with the Kolmogorov-Smirnov test and homogeneity of variances with Levene’s test. The *t*-test was used to evaluate differences between the two groups. As mentioned, the purpose of the first part of this study was to identify the optimal cut-off value of UA for a higher likelihood of developing MetS. In addition, this optimal cut-off value was calculated by ROC (MedCalc Software, Broekstraat, Mariakerke, Belgium) and used for grouping in the longitudinal analysis. In the second part of the study, the subjects with a higher UA level than that recorded in the first part of the study were observed to evaluate whether they had a higher risk of MetS, hypertension or type 2 DM after 10 years. Kaplan-Meier plots and the log rank test were performed to evaluate effect of the time on the occurrence of the future events between these two groups. Finally, both univariate and multivariate Cox regression analyses were performed to obtain the hazard ratios (HR) of both groups during the follow-up period. A p-value (two-sided) < 0.05 was considered to be significant. All statistical analyses were performed using SPSS software version 13.0 (SPSS Inc., Chicago, IL).

## Results

The baseline demographic data were shown in Table 1. There were 3,263 males with a mean age of 12.7 ± 1.7 years, 259 (7.9%) of whom had MetS. In addition, 2,485 females were also enrolled with a mean age of 12.8 ± 1.7 years, of whom 186 (7.5%) had MetS. Not surprisingly, those with MetS had significantly higher BMI, WC, SBP, SDP, FPG, LDL-C, TG and UA than those without MetS in both genders.

**Table 1:** Demographic data of the study subjects with and without metabolic syndrome at baseline.

	MetS (-)	MetS (+)	P value
Male			
n	3004	259	
Age (years)	12.7 ± 1.7	13.4 ± 1.5	< 0.001
BMI (kg/m^2^)	19.95 ± 4.08	25.58 ± 4.23	< 0.001
WC (cm)	68.1 ± 10.3	82.6 ± 10.6	< 0.001
SBP (mmHg)	109.8 ± 12.6	126.4 ± 13.1	< 0.001
DBP (mmHg)	60.0 ± 8.6	66.9 ± 9.6	< 0.001
FPG (mg/dl)	95.0 ± 8.4	100.0 ± 7.0	< 0.001
TC (mg/dl)	164.9 ± 29.0	169.3 ± 35.5	0.053
HDL-C (mg/dl)	57.0 ± 13.2	43.2 ± 10.5	< 0.001
LDL-C (mg/dl)	92.5 ± 25.2	98.8 ± 29.1	0.001
Triglyceride (mg/dl)	77.1 ± 35.9	136.4 ± 65.5	< 0.001
Uric acid (mg/dl)	6.7 ± 1.6	7.8 ± 1.7	< 0.001
Female			
n	2299	186	
Age (years)	12.8 ± 1.7	12.7 ± 1.7	0.276
BMI (kg/m^2^)	19.01 ± 3.42	23.26 ± 4.86	< 0.001
WC (cm)	63.2 ± 7.5	72.5 ± 9.4	< 0.001
SBP (mmHg)	104.9 ± 11.5	115.0 ± 15.0	< 0.001
DBP (mmHg)	58.8 ± 7.6	62.0 ± 8.9	< 0.001
FPG (mg/dl)	92.4 ± 10.0	98.8 ± 17.5	< 0.001
TC (mg/dl)	166.9 ± 27.3	171.3 ± 32.0	0.070
HDL-C (mg/dl)	58.2 ± 12.9	44.0 ± 8.6	< 0.001
LDL-C (mg/dl)	93.4 ± 24.3	98.1 ± 28.9	0.032
Triglyceride (mg/dl)	77.0 ± 31.1	146.3 ±69.0	< 0.001
Uric acid (mg/dl)	5.4 ± 1.2	6.0 ± 1.3	< 0.001

MetS(-), without metabolic syndrome; MetS(+), with metabolic syndrome; BMI, body mass index; WC, waist circumference; SBP, systolic blood pressure; DBP, diastolic blood pressure; FPG, fasting plasma glucose; TC, total cholesterol; HDL-C, high-density lipoprotein cholesterol; LDL-C, low-density lipoprotein cholesterol. Data are shown as mean ± SD

**Fig 2:**
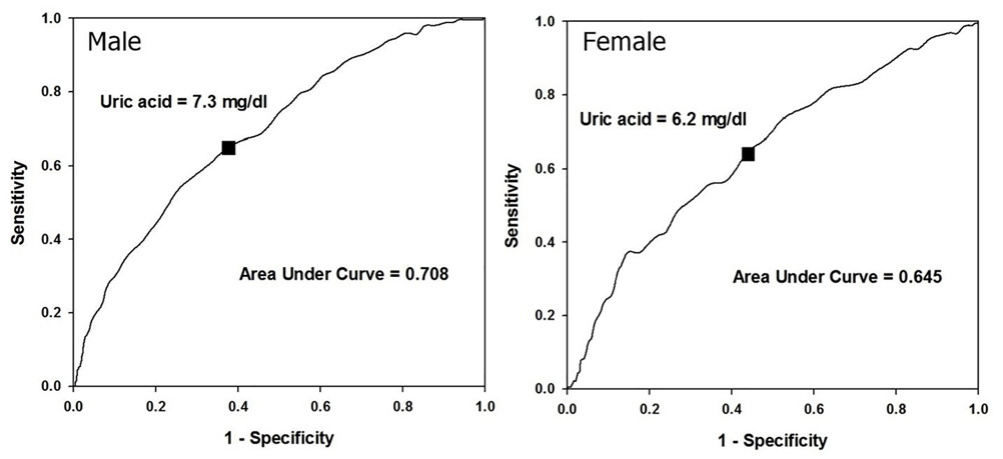
The optimal cut-off values of uric acid by receiver operating characteristic curves in both genders.

**Fig 3:**
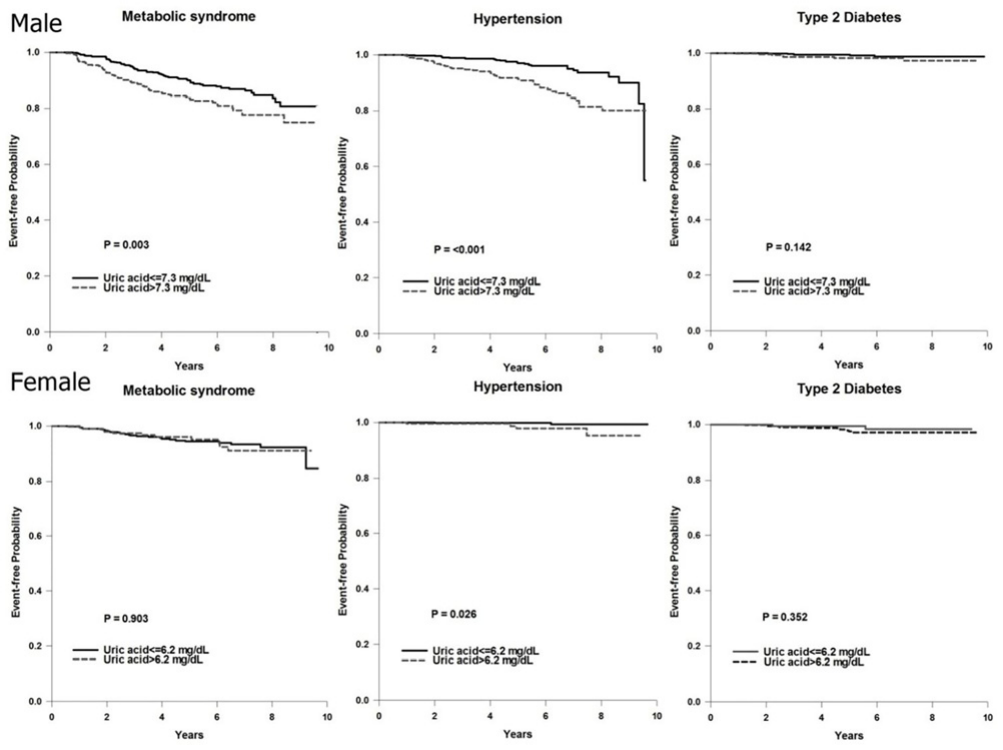
Kaplan-Meier plots of metabolic syndrome, hypertension and type 2 diabetes the uric acid level in both genders.

Fig 2 shows the results of the first part of the study, and the cutoff points derived from the ROC curves for male and female were 7.3 and 6.2 mg/dl, respectively. These values were then used as the grouping criteria to investigate the time effect and HR of the endpoints (Fig 3, Tables 2 and 3).

**Table 2:** Univariate hazard ratios of future metabolic syndrome, hypertension and type 2 diabetes by uric acid level and metabolic syndrome components.

	Male		Female	
	HR(95%CI)	P	HR(95%CI)	P
Metabolic syndrome				
UA > 7.6 mg/dl	1.658(1.186–2.318)	0.003	1.044(0.524–2.080)	0.903
WC > criteria*	4.282(2.713–6.759)	< 0.001	1.476(0.791–2.754)	0.221
BP > criteria*	1.264(0.736–2.170)	0.395	0.687(0.094–5.002)	0.711
FPG > 100 mg/dl	1.353(0.927–1.975)	0.117	1.252(0.491–3.193)	0.638
HDL-C < criteria*	0.992(0.595–1.653)	0.974	2.943(1.589–5.452)	0.001
TG > 150 mg/dl	1.141(0.599–2.172)	0.689	0.933(0.225–3.866)	0.924
Hypertension				
UA > 7.6 mg/dl	2.920(1.786–4.775)	< 0.001	5.522(1.021–30.174)	0.047
WC > criteria*	3.141(1.746–5.650)	< 0.001	4.442(0.519–38.043)	0.174
BP > criteria*	3.267(1.885–5.661)	< 0.001	25.519(5.141–126.676)	< 0.001
FPG > 100 mg/dl	0.929(0.517–1.671)	0.806	1.887(0.219–16.252)	0.563
HDL-C < criteria*	1.119(0.585–2.139)	0.734	1.793(0.361–8.900)	0.475
TG > 150 mg/dl	1.796(0.890–3.622)	0.102	9.625(1.744–53.112)	0.009
Type 2 Diabetes				
UA > 7.6 mg/dl	2.308(0.732–7.272)	0.153	0.496(0.110–2.239)	0.362
WC > criteria*	3.844(0.842–17.547)	0.082	1.009(0.339–3.003)	0.987
BP > criteria*	0.882(0.114–6.832)	0.904	2.292(0.298–17.628)	0.426
FPG > 100 mg/dl	2.547(0.808–8.029)	0.111	0.042(0.000–97.354)	0.422
HDL-C < criteria*	1.336(0.292–6.113)	0.709	0.845(0.260–2.744)	0.779
TG > 150 mg/dl	2.428(0.532–11.083)	0.252	0.046(0.000–1624.468)	0.564

HR, hazard ratio, CI, confidence interval; UA, uric acid; WC, waist circumference; BP, blood pressure; FPG, fasting plasma glucose; HDL-C, high-density lipoprotein cholesterol

*Criteria for WC according to the cut-off value by Sung et al[20]; criteria for BP were systolic BP > 130 mmHg or diastolic BP > 85 mmHg; criteria for HDL-C were < 40 mg/dl in males and < 50 mg/dl in females.

**Table 3:** Multivariate hazard ratios of future metabolic syndrome and hypertension by uric acid level and metabolic syndrome components.

	Male		Female	
	HR(95%CI)	P	HR(95%CI)	P
Metabolic syndrome				
UA > 7.6 mg/dl	1.402(1.006–1.987)	0.046	---	---
WC > criteria*	4.031(2.543–6.390)	0.000	---	---
BP > criteria*	---	---	---	---
FPG > 100 mg/dl	---	---	---	---
HDL-C < criteria*	---	---	2.943(1.589–5.452)	0.001
TG > 150 mg/dl	---	---	---	---
Hypertension				
UA > 7.6 mg/dl	2.337(1.411–3.748)	0.001	2.764(0.445–15.262)	0.256
WC > criteria*	2.376(1.316–4.212)	0.004	---	---
BP > criteria*	2.499(1.372–4.148)	0.001	16.344(3.454–89.575)	0.001
FPG > 100 mg/dl	---	---	---	---
HDL-C < criteria*	---	---	---	---
TG > 150 mg/dl	---	---	4.846(0.770–31.659)	0.097

HR, hazard ratio, CI, confidence interval; UA, uric acid; WC, waist circumference; BP, blood pressure; FPG, fasting plasma glucose; HDL-C, high-density lipoprotein cholesterol

*Criteria for WC according to the cut-off value by Sung et al[20]; criteria for BP were systolic BP > 130 mmHg or diastolic BP > 85 mmHg; criteria for HDL-C were < 40 mg/dl in males and < 50 mg/dl in females.

The results of the univariate analysis for the ratio of abnormal UA and other MetS components on the prediction of future MetS, type 2 diabetes and hypertension were shown in Table 2. Interestingly, a high level of UA was either the second or third best predictor of hypertension in both genders. Hypertension was the most important predictor for MetS in these adolescents, and a high level of UA only predicted future MetS in males. However, UA levels did not predict future type 2 DM in both genders. The results of the multivariate adjusted HR are shown in Table 3. The level of UA still had a significant relationship with the future occurrence of MetS and hypertension in males, however there was only a significant association in predicting hypertension but not MetS in the females.

Kaplan-Meier plots of the event-free proportion between the high- and low-UA groups who were followed for 10 years for the occurrence of MetS, hypertension and type 2 DM are shown in Fig 3. The males in the high-UA group had a significantly higher likelihood of having MetS and hypertension, however the females only had a higher likelihood of having hypertension. There were no differences in the likelihood of having type 2 DM in both genders.

## Discussion

Our results shows that the subjects with a high level of UA had a higher likelihood of having hypertension (both genders) and MetS (only males) after 10 years of follow-up. To the best of our knowledge, this is the first longitudinal study performed on this topic to include such a large cohort. Our results further consolidated the relationships between UA and MetS and hypertension.

### The relationship between UA and hypertension

The underlying mechanisms between UA and hypertension have been explored in animal studies, and hyperuricemic rats have been shown to develop hypertension due to preglomerular arteriolopathy [12,13]. In addition, hyperuricemia itself has been shown to activate the renin-angiotensin system, which increases sodium resorption [14]. The findings of the current study are in line with other major studies on serum levels of UA levels in both Caucasian and Asian adults [15,16]. Even in adolescents, similar results have also been reported in adolescents. For example, a recent National Health and Nutrition Examination Survey (NHANES) in the USA examined 6,036 adolescents aged 12 to 17 years from 1999 to 2006. The results showed that subjects with a high level of UA (> 5.5 mg/dL) had a 2-fold higher risk of having hypertension [17]. Even though that was a cross-sectional study, the present study further confirmed this relationship.

### The relationship between UA and MetS

The diagnosis of MetS in adults requires the presence of abdominal obesity plus the presence of two or more of the other components (elevated TG, low HDL, high cholesterol, high BP, and elevated plasma glucose). However, to date, there is no unified definition that can be used to assess the risk in children and adolescents. Moreover, consistent evidence-based cut-off points for the MetS components are still lacking. To address this issue, some researchers have suggested using modified definitions such as age-specific or by percentile [18–20]. We used the International Diabetes Federation definitions, ^7^ the most recognized guidelines, and our results were consistent with the results of the NHANES which also showed that 21% of the adolescents in the highest quartile of concentrations of UA had MetS compared to less than 1% in the lowest [21].

Since it is well-known that insulin resistance is at the core of MetS, the relationship between UA and MetS can be easily explained. Hyperuricemia and insulin resistance share bidirectional causal effects [22]. Hyperuricemia can cause endothelial dysfunction and inhibit nitric oxide bioavailability which leads to subsequent hyperinsulinemia. On the other hand, hyperinsulinemia can increase UA reabsorption in the proximal tubules which leads to hyperuricemia [23]. Thus, insulin resistance can be regarded as a bridge between obesity and hyperuricemia.

In the present study, we showed that levels of UA could predict the future occurrence of MetS only in males. This is partially contradictory to other major studies which reported a positive finding in both genders in adults [24]. The possible explanation for this discrepancy between males and females may be that UA metabolism is not uniform throughout puberty, and serum urate increases progressively as puberty advances [25]. One hypothesis is that fractional excretion of urate by the kidneys is less than that in adults [26,27]. In addition, there is a substantial increase in BMI during puberty. During adolescence, the weight of females is more than that of age-matched males, and the onset of puberty females is earlier. Furthermore, the increased level of estrogen in females during pregnancy has a hypouricemic effect [28]. These three factors may have contributed to our unusual results.

### The relationship between UA and type 2 DM

We did not find any predictive power between UA levels and future type 2 DM, which is not consistent with previous studies [29]. In Taiwan, an analysis of the Nationwide Health Insurance database from year 2000 to 2009 showed that the prevalence of type 2 diabetes was lowest in those under 19 years of age (about 0.06–0.08%) compared to all other age groups[30]. It is not surprising that UA does not relate to the incidence of diabetes considering the natural course of diabetes. Long before diabetes occurs, insulin resistance starts at a much earlier age, even probably from puberty. To compensate for this defect in glucose homeostasis, insulin secretion begins to increase. During this ‘compensated period’, the plasma glucose levels can still be maintained within a normal range. However, if fasting plasma insulin is measured in this period, it may be higher than normal as a result of β cell compensation. After decades, the β cells will eventually become exhausted and diabetes will be diagnosed through the elevation of FPG. Thus, due to the relatively young age and not fully developed insulin resistance, most adolescents may still have normal glucose metabolism, and this may explain why there was no significant relationship between UA and diabetes. Unfortunately, we did not have data on plasma insulin levels for this cohort so we were unable to evaluate the relationship between UA and insulin resistance.

There were several limitations to this study. First, the study cohort was selected from a health check-up center from 1999 to 2008, and there might have been substantial changes in the incidence of obesity and MetS from 2008 to now. However, we still believed that since our purpose was to evaluate the role of UA, the time of data collection might not be important. Second, the study cohort was purely ethnical Chinese. Thus, extrapolating our results to other ethnic groups should be done with caution. Finally, we did not have data on plasma insulin levels, and further studies should include this parameter.

## Conclusions

Adolescent males with a high UA level (≥ 7.3 mg/dL) had a higher risk of hypertension and MetS after 10 years of follow-up. However, significance was only noted between UA and hypertension but not MetS in adolescent females with a UA level ≥ 6.2 mg/dL. The levels of UA did not affect the future likelihood of having diabetes in both genders. The role of UA in MetS and hypertension in adolescents should not be overlooked.

## Supporting Information

S1 Dataset Raw data used in this study. (XLSX)
